# Data-Driven GENERIC Modeling of Poroviscoelastic Materials

**DOI:** 10.3390/e21121165

**Published:** 2019-11-28

**Authors:** Chady Ghnatios, Iciar Alfaro, David González, Francisco Chinesta, Elias Cueto

**Affiliations:** 1 Mechanical Engineering Department, Notre Dame University-Louaizé, Zouk Mosbeh P.O. Box 72, Lebanon; 2 Aragon Institute of Engineering Research, Universidad de Zaragoza, Edificio Betancourt, Maria de Luna, s.n., 50018 Zaragoza, Spain; iciar@unizar.es (I.A.); ecueto@unizar.es (E.C.); 3 ESI Chair @ ENSAM Arts et Metiers Institute of Technology, 151 Boulevard de l’Hôpital, F-75013 Paris, France; Francisco.Chinesta@ensam.eu

**Keywords:** soft materials, biphasic materials, hydrogel, data-driven, GENERIC, modeling

## Abstract

Biphasic soft materials are challenging to model by nature. Ongoing efforts are targeting their effective modeling and simulation. This work uses experimental atomic force nanoindentation of thick hydrogels to identify the indentation forces are a function of the indentation depth. Later on, the atomic force microscopy results are used in a GENERIC general equation for non-equilibrium reversible–irreversible coupling (GENERIC) formalism to identify the best model conserving basic thermodynamic laws. The data-driven GENERIC analysis identifies the material behavior with high fidelity for both data fitting and prediction.

## 1. Introduction

Thanks to the recent progress in simulation technology and computing power, the mechanical behavior of biological tissues is nowadays one of the most active research topics. However, many biological tissues are biphasic by nature, which renders their effective modeling and simulation a challenging issue, even with the impressive progress achieved recently [1]. Human cartilage, for example, is a biphasic material, where the fluid pressurization is believed to be the main load-carrying phenomenon [2]. Moreover, the microstructure and the fluid-solid interactions in such materials is complex and not fully understood [3,4]. The modeling of biphasic materials is, however, mandatory to design effective replacements of human soft tissues as well as understanding their behavior [5].

Different efforts are undertaken to model soft materials. In [5], the authors attempt to define a viscoelastic model based on only three parameters identified experimentally, using a pure solid mechanics approach. Other works aim to model the contact and lubrication phenomenon when using soft materials in contact mechanics, similarly to human body lubricated contact [6]. In [6], an interface element is developed and identified to transmit contact efforts from contact bodies into the lubrication fluid, soft material film. A study of cutting behavior of soft material films is performed in [7] using energy methods, however, assuming a non-linear hyperelastic neoprene rubber-like material. In [1], the authors model the soft material indentation using a biphasic approach, combining an elastic solid behavior and a fluid pressurization one. Indentation of soft materials is also studied in [8], where the authors performed a theoretical and finite element study using Hertzian contact and generalizing for large hyperelastic deformation including material non-linearities. Suitable nonlinear compressible material model for soft materials is also derived in [9], with its parameters identified for lung parenchyma using experimental results and inverse analysis.

All the aforementioned studies use experimental results to tune previously defined models, either using finite element simulation or analytical derivations. However, the derived models will always lack the exact truth due to the missing knowledge or information, and the errors involved in experimental measurements. Moreover, models derived to reproduce a given test situation may work pretty well in that specific test. However, their predictive ability in different loading cases, boundary conditions, or other basic variations in the problem, may not be as accurate as desired. This fact has lead to the development of data-driven models to overcome the need to predefine a “model”, as well as hybrid-twins models aiming to correct established models with experimental results [10]. Data-driven models aim to create models straight out of abundant data, either through regressions/optimized regressions [11], or through the use of artificial neural networks [12]. While hybrid-twin models aim to perform an error correction on well-established models, aiming to preserve the known physics in the model, while accounting for the ignorance or lack of information [10,13]. Such a trend is now colonizing more research fields and soft materials are not an exception [14,15]. For example, Ref. [14] trained a neural network to simulate later in real-time the response of human soft tissues in a surgical context. In [15], the authors leverage machine learning techniques along with classical mechanics of materials models to create a hybrid modeling of soft materials.

Despite being popular nowadays, data-driven models and hybrid ones sometimes can violate the basic established principles of physics, like mass and energy conservation, entropy production, etc., especially when the data sample size is not large enough and/or contains multiple outliers. In fact, no physical constraint is currently integrated in machine learning techniques to alleviate the possibility of violating the basic principles of thermodynamics. Recent efforts aim to create a physically informed neural network for supervised learning, through integrating the physical differential equation error in the neural network optimization [16,17]. An alternative approach to design models from data while conserving physical constraints is the GENERIC formalism [18]. In fact, GENERIC stands for «general equation for non-equilibrium reversible–irreversible coupling». The GENERIC formalism originally came from modeling rheological behavior of complex fluids, and emerged gradually from the treatment of different cases [19,20] as stated in [21,22]. Later on, a detailed publication of the approach was given in [23].

Recently, the GENERIC formalism is used to derive data-driven modeling, while preserving basic thermodynamics laws [18]. Such an approach is successfully applied to derive hyperelastic materials behavior from data measurements [18]. GENERIC is also used to identify the best suitable model of the experimental results using only data values [18]. The GENERIC formalism establishes a complete and general equation of motion of the system under reversible and/or irreversible conditions of the system [23]. Such an approach is leveraged to identify thermodynamically consistent data-driven models out of experimental results, without setting any a priori assumptions, except the basic laws of thermodynamics.

This work consists of a first attempt to model soft materials, hydrogels, for instance, using the GENERIC formalism and experimental data. This work aims to formulate a new constitutive model using experimental data and the conservation of thermodynamic quantities. The experimental setup consists of a unidirectional nanoindentation test with atomic force microscopy, using a spherical rigid indenter. The experimental device measures the displacement and the spring back force at the indenter. The indented specimen is a highly porous hydrogel medium, which is a material of high interest currently [24]. In fact, hydrogels are biphasic composite soft materials used, among others, for drug cell encapsulation controlling drug release, contact lenses, cartilage. Ongoing efforts are made to simulate and characterize these materials [24,25,26]. However, the ongoing works all starts by assuming a physical model for the material in question.

The work starts with a review of the experimental indentation process at hand, giving the reaction forces in a thick hydrogel poroviscoelastic material, as a function of the process input parameters: the indentation depth and velocity of the indenter. Later on, a modeling of the process is performed and then the GENERIC formalism used in this work is detailed along with the identified model. Finally, the numerical results are illustrated and discussed.

## 2. Numerical Modeling of the Indentation Process

### 2.1. The Experimental Procedure

**Figure 1:**
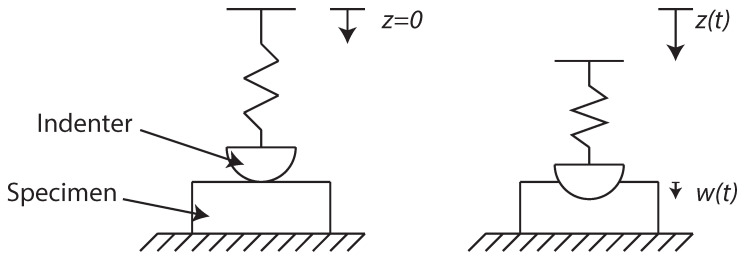
The reference for z=0 is taken at the point of contact of the indenter with the specimen, w(t) is the penetration depth of the probe into the specimen.

In this section, we review the experimental setup used to identify the mechanical behavior of a thick hydrogel. The experiment uses atomic force microscopy nanoindentation (AFM nanoindentation) to identify the mechanical response of a hydrogel. AFM is usually modeled as a cantilever beam supporting a semi-spherical indenter of radius ρ=36μm. In turn, the cantilever beam is modeled as a spring of stiffness k=2.88 N/m, as illustrated in Figure 1.

The specimen indentation depth is w(t), while the displacement at the base is z(t). The experimental setup identifies z(t) using optical sensors and the reaction force *F* at the base of the spring, which corresponds to the force in the spring. Therefore one may easily write:

(1) $$w=z-\frac{F}{k}.$$

**Figure 2:**
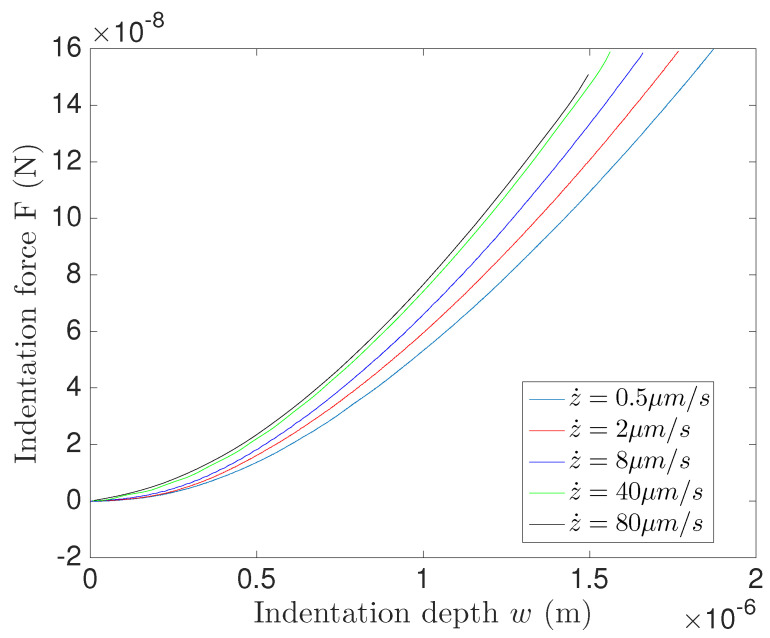
The experimental atomic force microscopy nanoindentation (AFM) nanoindentation reaction force in the spring F(t) as a function of the indentation depth w(t).

The experiment is repeated for five different indentation rates, $\dot{z}$, equal to 0.5μm/s, 2μm/s, 8μm/s, 40μm/s and 80μm/s. The experimental results are illustrated in Figure 2, which provides real experimental data obtained using atomic force microscopy (AFM) non-indentation. The experiments were performed at room temperature or $22{\;}^{{}^{\circ}}$C ± 1 ${}^{{}^{\circ}}$C. The normal spring constant of the cantilever was measured using the thermal noise method before attaching the colloidal microsphere indenter. The indenter is a silica microsphere glued with UV-curable glue (Norland optical adhesive 63) to the end of the tipless cantilever by means of a home-built micromanipulator. The exact approach rates $\dot{z}\left(t\right)$ were measured using a Z-piezo sensor to control the approach of the probe as much as possible. The experimental setup measures *z* and *F* at every time step Δt=0.5 ms. One may note that the indentation depth does not exceed 1.8μm as shown in Figure 2, and therefore we can approximate the spherical indenter by a flat one considering the radius of curvature of the indenter ρ as very large with respect to the indentation depth. Thus the indentation area can be approximated as $A=\pi R^{2}$ with *R* the indentation radius defined as:

(2) $$R=\sqrt{\rho^{2}-{\left(\rho -w\right)}^{2}}.$$

Moreover, we note that the initial hydrogel specimen height $H_{0}$ is large enough to avoid any substrate effect during the indentation. For instance, in the tested specimen, $H_{0}=6$ mm, more than 1000 times the maximum reached indentation depth.

### 2.2. GENERIC Formalism

Under the framework of non-equilibrium thermodynamics, the GENERIC formalism establishes a completely general equation of the dynamics of a system under reversible and irreversible conditions, as dictated by the evolution of energy and entropy, respectively [22,23,27]. Models constructed using the GENERIC formalism preserve the symmetries of the system and therefore guarantee the conservation of energy and the increase of entropy.

The GENERIC structure of the evolution equations for an arbitrary problem is

(3) $${\dot{z}}_{t}=L\left(z_{t}\right)\nabla_{z}E\left(z_{t}\right)+M\left(z_{t}\right)\nabla_{z}S\left(z_{t}\right),\;\;z\left(0\right)=z_{0},$$

where:

1. [$z_{t}$] is the vector of state variables of the problem at time *t*. The choice of $z_{t}$ is irrelevant in the sense that different sets of $z_{t}$ lead to different GENERIC formalisms, all of them thermodynamically consistent. However, $z_{t}$ should contain variables—such as position, momentum, stress, energy, or entropy, for instance—able enough to evaluate the energy conservation and the dissipation therms.
***L***  is the so-called Poisson matrix and will be responsible for the reversible (Hamiltonian) part of the evolution of the system.
*E*  represents the energy of the system, as a function of its particular state at time *t*, $z_{t}$.
***M***  represents the friction matrix, responsible for the irreversible part of the evolution of the system.
*S*  represents the entropy of the system for the particular choice of variables z.

Equation (3) is supplemented with the complementary degeneracy conditions, i.e.,

(4) $$\begin{array}{cc} L\left(z\right)\cdot \nabla_{z}S\left(z\right) & =0,\\ M\left(z\right)\cdot \nabla_{z}E\left(z\right) & =0. \end{array}$$

In what follows, if there is no risk of confusion, and for the sake of readability, we will omit the subscript *z* in $\nabla_{z}$.

By choosing L skew-symmetric and M symmetric, positive semi-definite, one ensures the conservation of energy:

(5) $$\dot{E}\left(z\right)=\nabla E\left(z\right)\cdot \dot{z}=\nabla E\left(z\right)\cdot L\left(z\right)\nabla E\left(z\right)+\nabla E\left(z\right)\cdot M\left(z\right)\nabla S\left(z\right)=0,$$

and the fulfillment of the second principle of thermodynamics:

(6) $$\dot{S}\left(z\right)=\nabla s\left(z\right)\cdot \dot{z}=\nabla S\left(z\right)\cdot L\left(z\right)\nabla E\left(z\right)+\nabla S\left(z\right)\cdot M\left(z\right)\nabla S\left(z\right)\geq 0.$$

### 2.3. Data-Driven Characterization of the GENERIC Description of a Hydrogel

The objective is to model the biphasic hydrogels using GENERIC formalisms, thus to obtain the GENERIC Equation (3) for the experimental data. In [18,28] an approach has been developed by the authors so as to obtain a numerical description—by means of manifold learning techniques—of the different constituents of the GENERIC equation.

Since data $z_{i}$ are obtained at discrete time increments, Equation (3) is first discretized in time,

(7) $$\frac{z_{n+1}-z_{n}}{\Delta t}=L\left(z_{n}\right)\mathrm{DE}\left(z_{n}\right)+M\left(z_{n}\right)\mathrm{DS}\left(z_{n}\right),$$

where, for simplicity, we denote $z_{n+1}=z_{t+\Delta t}$ and where L and M are the discrete version of the Poisson and friction operators, respectively. Also, DE and DS represent the discrete gradients. In general, matrix L is constant over the process, while matrix M is frequently a function of z.

The set of variables chosen for each $z_{i}$ are the specimen indentation depth w(t), the specimen indentation velocity $v\left(t\right)=\dot{w}\left(t\right)$ and the average normal stress at the indentation point σ(t) at time $t_{i}=i\times \Delta t$. Also, in this paper, we assume L known, being:

(8) $$L=\left(\begin{array}{ccc} 0 & 1 & 0\\ -1 & 0 & 0\\ 0 & 0 & 0 \end{array}\right).$$

As can be seen in [28], different definitions of z, L and M can be done, and also the particular structure of L can be considered itself as an unknown. The ones selected here have shown the best convergence for this particular problem. Furthermore, the discrete gradients are discretized in a piecewise linear, finite element, manner:

(9) $$\mathrm{DE}\left(z_{n}\right)=A\cdot z_{n},\;\mathrm{DS}\left(z_{n}\right)=B\cdot z_{n},$$

where A and B can be time dependent operators or constant with respect to the time. For the selected problem, operators were shown to be almost constant. Therefore, we decided to perform the regression procedure within a single step. The proposed algorithm will thus consist in solving the following minimization problem:

(10) $$\mu^{*}=\left\{A,M,B\right\}=\underset{\mu }{arg min}||z\left(\mu \right)-z^{\exp}\left|\right|,$$

subjected to:

(11) $$\begin{array}{cc} L\cdot B\cdot z(\mu ) & =0,\\ M\cdot A\cdot z(\mu ) & =0, \end{array}$$

with z(μ) given by Equation (7) and $z^{\exp}$ all the experimental results of each experiment.

The just-introduced procedure must not be seen as a model fitting procedure. Indeed, the number of values to determine is much higher than the usual number of parameters in a suitable model. What we seek with this procedure is a method of machine learning the GENERIC expression of the problem. The result is the numerical value of these GENERIC building blocks and not the particular values of any model parameter. Those are considered constant for each experiment, although a piece-wise linear variation along time is equally possible, in general.

Once the minimization problem has been solved, and given an initial $z_{0}$ value of the variable at the beginning of the experiment, it is possible to reconstruct the solution for each experiment, in order to check the accuracy of the method:

(12) $$\left(\begin{array}{c} w_{n+1}\\ {\dot{w}}_{n+1}\\ \sigma_{n+1} \end{array}\right)=\left(\begin{array}{c} w_{n}\\ {\dot{w}}_{n}\\ \sigma_{n} \end{array}\right)+\Delta t\left(L\cdot A+M\cdot B\right)\left(\begin{array}{c} w_{n}\\ {\dot{w}}_{n}\\ \sigma_{n} \end{array}\right).$$

### 2.4. Equivalence With Traditional Ways of Phenomenological Model Fitting

For practitioners used to employ experimental results for constitutive law fitting purposes, the procedure outlined above could seem intricate and unclear. Particularly, regression of the terms in Equation (3) could somehow obscure the true philosophy behind the suggested method.

Note, however, that some terms in Equation (3) should seem familiar to us. Noteworthy, the term ∇E(z) represents the usual form of deriving a constitutive law in a hyperelastic framework. In turn, the term ∇S(z) introduces a second potential, of dissipative nature, taking care of the viscous terms in the constitutive law.

From a practical point of view, the development of finite element time integration schemes deriving directly from GENERIC expressions is something already developed by different authors, particularly I. Romero and coworkers. Equation (3) could be employed advantageously to derive time integration schemes directly that conserve energy and dissipate entropy, showing great robustness from the numerical point of view, even for low orders. The interested reader is referred to [29,30,31] and references therein for details.

## 3. Results

### 3.1. GENERIC Model

The experimental measurements are taken every Δt=0.015 s, displacements are measured in micrometers μm, velocities in micrometers per second μm/s and stresses in nanoNewtons (nN) per square micrometer nN/${\left(\mu m\right)}^{2}$. A regression for A, M, and B is performed using different sets of measurements at different indentation rates. The experiment is performed on two different sets of materials.

**Figure 3:**
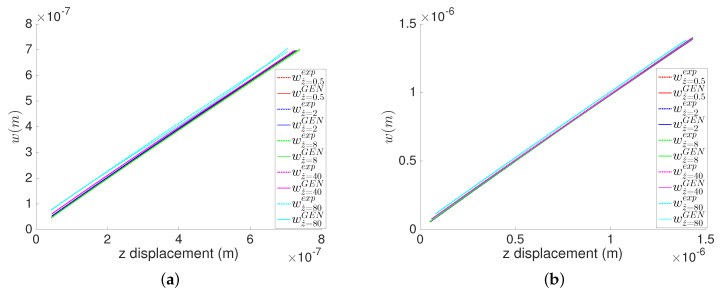
Indentation depth at different indentation rates for the MICA silicate and polyethylene hydrogels. Measurements are taken at constant time step Δt=15 ms. (**a**) MICA silicate fibers; (**b**) Polyethylene fibers.

Figure 3a,b show the indentation depth *w* as a function of the number of measurements taken from the beginning of the experiment, which is proportional to the time *t* since measurements are taken every 15 ms. We can clearly see an excellent comparison of the derived GENERIC model with the experimental indentation depth, with an average relative error of 0.4%.

**Figure 4:**
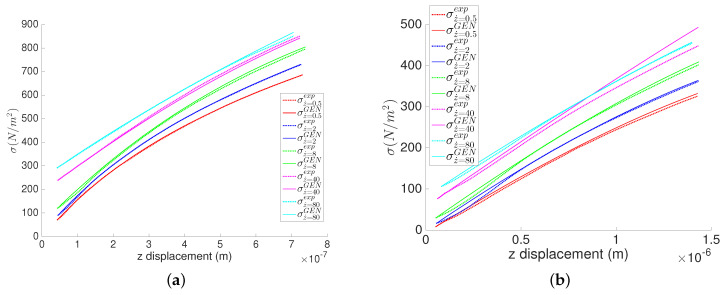
Normal stress during the indentation of the MICA silicate and polyethylene hydrogels at different indentation speed. Measurements are taken at constant time step Δt=15 ms. (**a**) MICA silicate fibers; (**b**) Polyethylene fibers.

Figure 4a,b show for both materials the experimental values of the state variables forming the stress σ value for different indentation velocities, along with their corresponding identified GENERIC models, obtained with the GENERIC regression. It is worth mentioning the high accuracy obtained. The stress results illustrate low relative error with respect to the experimental results.

**Figure 5:**
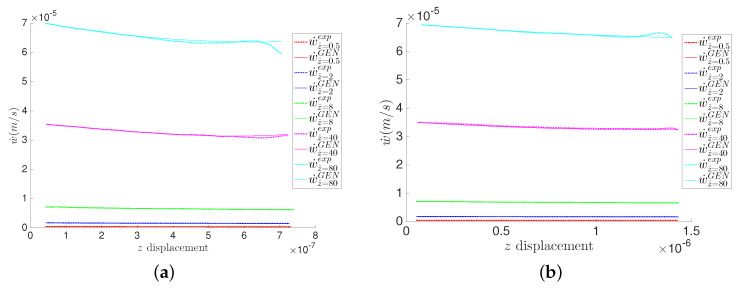
Indenter velocity $\dot{w}$ in the MICA silicate and polyethylene hydrogels at different $\dot{z}$. Measurements are taken at constant time step Δt=15 ms. (**a**) MICA silicate fibers; (**b**) Polyethylene fibers.

Using the generic formalism, we can also show the indenter velocity $\dot{w}$ obtained from the model and the experimental data through time derivation of the obtained data points *w* using explicit Euler’s derivation. Figure 5a,b show, for both materials, the experimental values of the indenter velocity $\dot{w}$ for different $\dot{z}$, along with their corresponding identified GENERIC models, obtained with the GENERIC regressions. In Figure 5, we can clearly see experimental artifacts of the velocity $\dot{w}$ at high indentation rates $\dot{z}$ and near the end of the experiment, for both materials. This is mainly due to the low machine precision at high speeds and potentially induced vibration of the cantilever arm.

**Figure 6:**
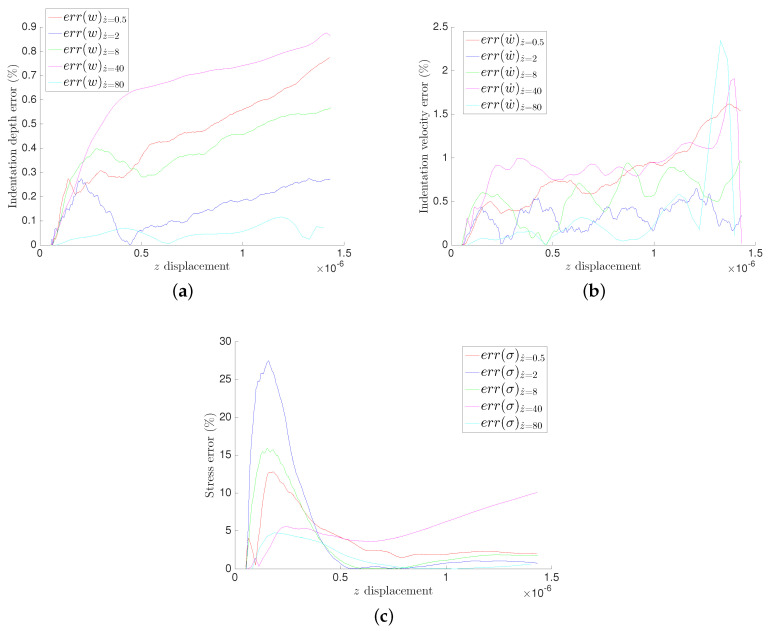
Relative error in the polyethylene fibers hydrogel for the three considered quantities of interest. (**a**) Indentation depth *w*; (**b**) Indeter’s velocity $\dot{w}$; (**c**) Average stress.

**Figure 7:**
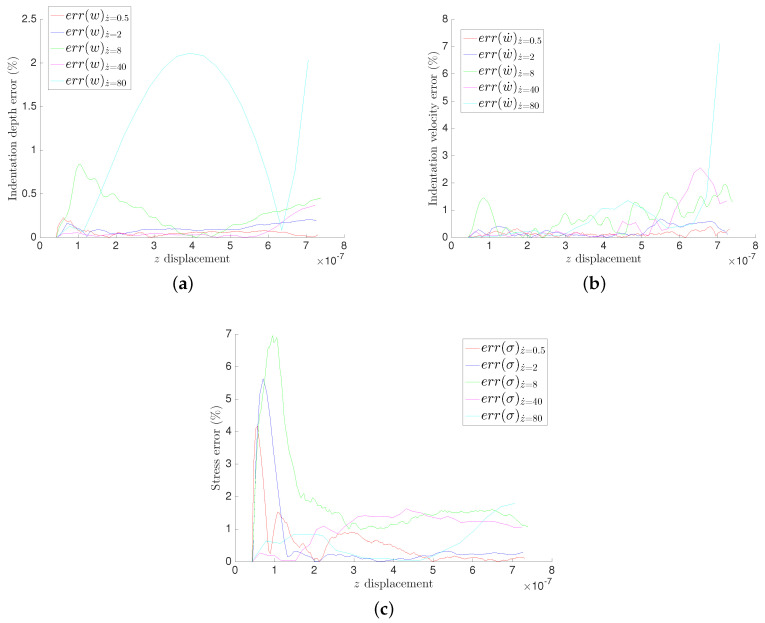
Relative error in the MICA silicate fibers hydrogel for the three considered quantities of interest. (**a**) Indentation depth *w*; (**b**) Indeter’s velocity $\dot{w}$; (**c**) Average stress.

The relative errors for different GENERIC formalism modeling, for the three quantities of interests, are illustrated in Figure 6 and Figure 7 for the MICA silicate and polyethylene fibers hydrogels respectively.

All experiments have low relative errors in general. We can clearly see negligible relative error for the indentation depth *w* for both materials in Figure 6a and Figure 7a. We can also identify very low relative errors on the indenter’s velocity $\dot{w}$ in Figure 6b and Figure 7b, except by the end of the experiments at $\dot{z}=40\;\mu$m/s and $\dot{z}=80\;\mu$m/s, which is explained by the experimental artifacts seen by the end of the experiments. The stresses exhibit the highest values of the relative error while being in acceptable ranges eventually. The high relative errors at the beginning of the experiments illustrated in Figure 6c and Figure 7c are explained by the numerical and experimental amplified errors by the derivation of the results, since computing the velocities requires a numerical derivation of the experimental measurements.

Table 1 and Table 2 illustrate the average relative errors for the MICA silicate and polyethylene indentations experiments respectively, calculated using:

(13) $$\mathrm{error}=\frac{\sum_{i=1}^{N}∥\frac{z_{i}^{\exp}-z_{i}^{\mathrm{GEN}}}{z_{i}^{\exp}}∥}{N},$$

where *N* is the number of measurements in the experiment using a given indentation rate.

### 3.2. Predictive Capabilities for New Experimental Results

In this section we use the aforementioned GENERIC regression to predict the experimental results for an indentation velocity $\dot{z}=2\;\mu m/s$, while not being present in the training database. For that aim, we will use the GENERIC formalism matrices A, M and B obtained at $\dot{z}=0.5\;\mu m/s$ and $\dot{z}=8\;\mu m/s$. Using these results, we predict the matrices at $\dot{z}=2\;\mu m/s$ using a linear interpolation approach. For instance:

(14) $$A_{@2\mu m/s}=\frac{A_{@0.5\mu m/s}\times 6.5+A_{@8\mu m/s}\times 1.5}{8}.$$

**Figure 8:**
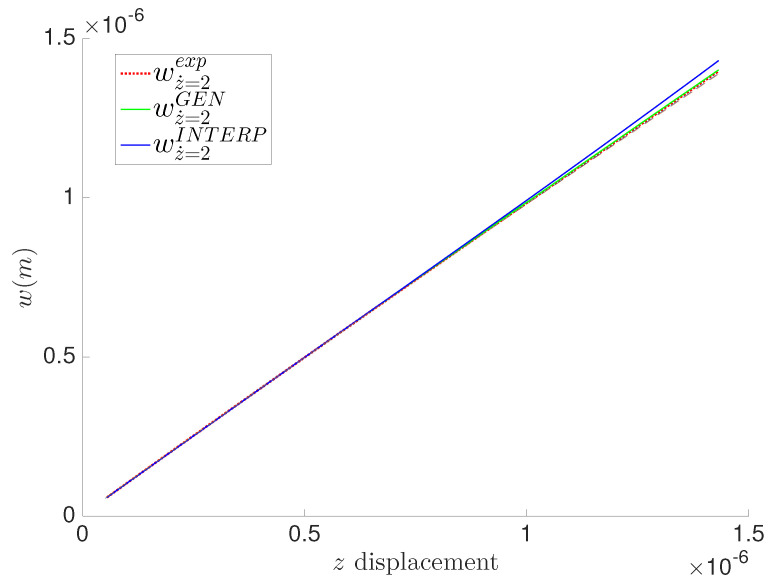
Prediction of the indentation depth at $\dot{z}=0.2\;\mu$m/s for the polyethylene fibers hydrogel.

**Figure 9:**
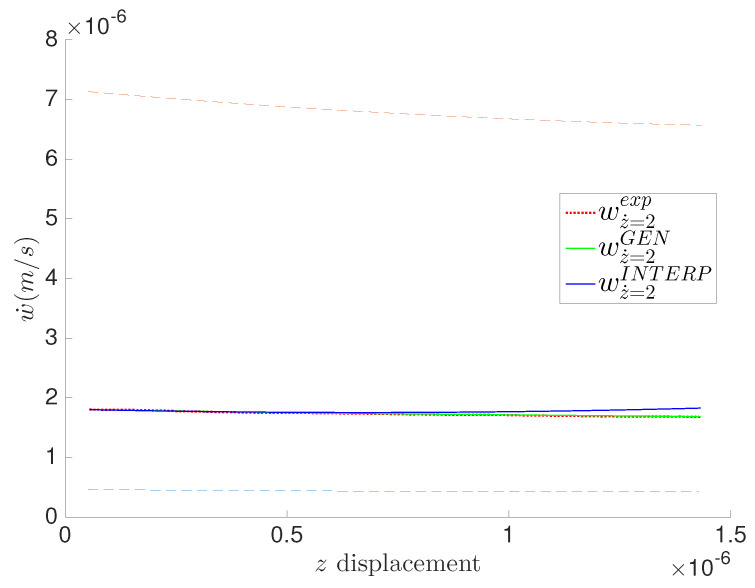
Prediction of the indenter’s velocity $\dot{w}$ at $\dot{z}=0.2\;\mu$m/s for the polyethylene fibers hydrogel.

**Figure 10:**
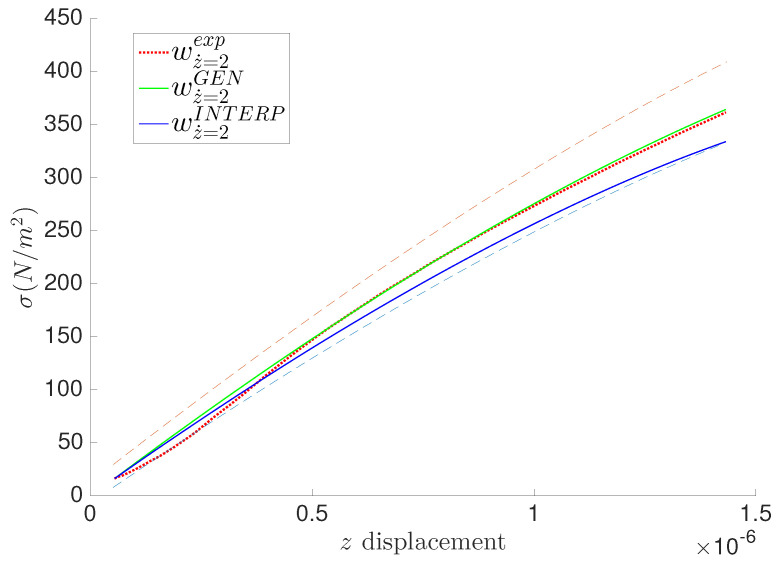
Prediction of the average stress at $\dot{z}=0.2\;\mu$m/s for the polyethylene fibers hydrogel.

Using the linear interpolation approach, we predict the GENERIC formalism matrices. Eventually, using a more accurate interpolation scheme is an option for interested users (Kriging for example). The prediction results are illustrated in Figure 8, Figure 9 and Figure 10. The results show good predictions bounded between the two fitted quantities of interest at $\dot{z}=0.5\;\mu$m/s and $\dot{z}=8\;\mu$m/s illustrated in large dashed lines in Figure 8, Figure 9 and Figure 10.

## 4. Discussion

This work consists of a first attempt to explore the possibilities of using the GENERIC formalism with data-driven identification, to model the indentation of thick hydrogels. The results show a good match with the experimental data. The use of three different matrices to identify in the model gives an advantage and flexibility of identifying a model with 27 parameters. The 27 parameters (3 matrices each containing 9 values) are eventually enough to reproduce the best behavior of the material. Indeed, not all the identified parameters are independent. For instance, the symmetry of the M matrix and the skew-symmetry of the L matrix reduces the number of independent variables to 18. Other restrictions may reduce even further the number of independent variables. Classical modeling of the indentation is possible, either using solid mechanics approach [32], or a combination of solid and fluid phases [1].

Different matrices A, M, and B are identified for different indentation velocities. This fact shows a highly non-linear behavior of the thick hydrogel in question. To simulate the behavior of the thick hydrogel at an indentation rate different than the studied ones, an SSL-PGD interpolation, for example, can be performed, for each one of the 27 identified parameters, among many nonlinear interpolation techniques [33]. The result of the identified matrices is illustrated in Appendix A and Appendix B for the two selected hydrogels.

The choice of using the GENERIC formalism appears to be a suitable approach for identifying models with challenging mechanical behavior but without prior exact knowledge of the constitutive equations. It also shows good predictive abilities for unfitted experimental results, as illustrated in Section 3.2.

## 5. Conclusions

In this work, we investigate the possibility of using the GENERIC formalism for modeling non-trivial material behavior. For instance, the work focuses on modeling thick hydrogels using only the conservation of thermodynamic laws. The results show a good correlation with the experimental ones for different experiments. The predictive ability of the model is illustrated in Section 3.2, where linear interpolation of the model matrices is shown to suffice for predicting new experimental models. Extrapolation of the results requires further thorough investigation and developments to yield good results.

## Figures and Tables

**Table 1:** Total error (%) during the indentation of the MICA hydrogel.

Variable	$\dot{z}=0.5\;\mu$m/s	$\dot{z}=2\;\mu$m/s	$\dot{z}=8\;\mu$m/s	$\dot{z}=40\;\mu$m/s	$\dot{z}=80\;\mu$m/s	Average Error
*w*	0.06	0.11	0.29	0.07	1.1	0.33
$\dot{w}$	0.14	0.24	0.77	0.53	0.85	0.51
σ	0.52	0.55	1.91	1.02	0.57	0.91

**Table 2:** Total error (%) during the indentation of the polyethylen hydrogel.

Variable	$\dot{z}=0.5\;\mu$m/s	$\dot{z}=2\;\mu$m/s	$\dot{z}=8\;\mu$m/s	$\dot{z}=40\;\mu$m/s	$\dot{z}=80\;\mu$m/s	Average Error
*w*	0.36	0.16	0.39	0.64	0.05	0.34
$\dot{w}$	0.8	0.33	0.55	0.91	0.34	0.59
σ	3.88	4.71	3.45	5.46	1.4	3.78

## Appendix A. Polyethylene Hydrogels Matrices

In this appendix we illustrate the final identified matrices by the considered algorithm for the Polyethylene hydrogels:

$\dot{z}=0.5\;\mu m/s$	$L=\left[\begin{array}{ccc} 0 & 1 & 0\\ -1 & 0 & 0\\ 0 & 0 & 0 \end{array}\right]$	$A=\left[\begin{array}{ccc} 0.020 & -0.25 & -0.010\\ -0.11 & 1.3 & 0.31\\ -0.090 & 1.05 & 0.30 \end{array}\right]$

	$M=\left[\begin{array}{ccc} 1 & 1 & -1\\ 1 & 1 & -1\\ -1 & -1 & 1 \end{array}\right]$	$B=\left[\begin{array}{ccc} 0 & 0 & 0\\ 0 & 0 & 0\\ -0.11 & 0.30 & 0.33 \end{array}\right]$

$\dot{z}=2\;\mu m/s$	$L=\left[\begin{array}{ccc} 0 & 1 & 0\\ -1 & 0 & 0\\ 0 & 0 & 0 \end{array}\right]$	$A=\left[\begin{array}{ccc} 0.92 & -0.18 & -3.19\\ -0.4 & 1.32 & 0.92\\ 0.52 & 1.14 & -2.27 \end{array}\right]$

	$M=\left[\begin{array}{ccc} 1 & 1 & -1\\ 1 & 1 & -1\\ -1 & -1 & 1 \end{array}\right]$	$B=\left[\begin{array}{ccc} 0 & 0 & 0\\ 0 & 0 & 0\\ -0.39 & 0.32 & 0.91 \end{array}\right]$

$\dot{z}=8\;\mu m/s$	$L=\left[\begin{array}{ccc} 0 & 1 & 0\\ -1 & 0 & 0\\ 0 & 0 & 0 \end{array}\right]$	$A=\left[\begin{array}{ccc} 0.29 & 0.35 & -7.16\\ -2.36 & 1.32 & 6.4\\ -2.07 & 1.67 & -0.77 \end{array}\right]$

	$M=\left[\begin{array}{ccc} 1 & 1 & -1\\ 1 & 1 & -1\\ -1 & -1 & 1 \end{array}\right]$	$B=\left[\begin{array}{ccc} 0 & 0 & 0\\ 0 & 0 & 0\\ -2.36 & 0.32 & 6.43 \end{array}\right]$

$\dot{z}=40\;\mu m/s$	$L=\left[\begin{array}{ccc} 0 & 1 & 0\\ -1 & 0 & 0\\ 0 & 0 & 0 \end{array}\right]$	$A=\left[\begin{array}{ccc} -191.54 & 3.59 & 267.13\\ -20.22 & 1.23 & 61.42\\ -211.76 & 4.82 & 328.55 \end{array}\right]$

	$M=\left[\begin{array}{ccc} 1 & 1 & -1\\ 1 & 1 & -1\\ -1 & -1 & 1 \end{array}\right]$	$B=\left[\begin{array}{ccc} 0 & 0 & 0\\ 0 & 0 & 0\\ -20.31 & 0.23 & 61.54 \end{array}\right]$

$\dot{z}=80\;\mu m/s$	$L=\left[\begin{array}{ccc} 0 & 1 & 0\\ -1 & 0 & 0\\ 0 & 0 & 0 \end{array}\right]$	$A=\left[\begin{array}{ccc} -143.65 & 5.04 & -43.91\\ -3.09 & 1.31 & -0.62\\ -146.74 & 6.35 & -44.53 \end{array}\right]$

	$M=\left[\begin{array}{ccc} 1 & 1 & -1\\ 1 & 1 & -1\\ -1 & -1 & 1 \end{array}\right]$	$B=\left[\begin{array}{ccc} 0 & 0 & 0\\ 0 & 0 & 0\\ -2.99 & 0.31 & -1.23 \end{array}\right]$

## Appendix B. MICA Silicate Hydrogels Matrices

In this appendix we illustrate the final identified matrices by the considered algorithm for the MICA silicate hydrogels:

$\dot{z}=0.5\;\mu m/s$	$L=\left[\begin{array}{ccc} 0 & 1 & 0\\ -1 & 0 & 0\\ 0 & 0 & 0 \end{array}\right]$	$A=\left[\begin{array}{ccc} -0.81 & -1.6 & 1.43\\ 0.73 & 2.74 & -1.41\\ -0.080 & 1.13 & 0.013 \end{array}\right]$

	$M=\left[\begin{array}{ccc} 1 & 1 & -1\\ 1 & 1 & -1\\ -1 & -1 & 1 \end{array}\right]$	$B=\left[\begin{array}{ccc} 0 & 0 & 0\\ 0 & 0 & 0\\ 0.74 & 1.74 & -1.43 \end{array}\right]$
$\dot{z}=2\;\mu m/s$	$L=\left[\begin{array}{ccc} 0 & 1 & 0\\ -1 & 0 & 0\\ 0 & 0 & 0 \end{array}\right]$	$A=\left[\begin{array}{ccc} -0.53 & -1.03 & 1.57\\ 2.92 & 2.81 & -5.23\\ 2.4 & 1.79 & -3.66 \end{array}\right]$

	$M=\left[\begin{array}{ccc} 1 & 1 & -1\\ 1 & 1 & -1\\ -1 & -1 & 1 \end{array}\right]$	$B=\left[\begin{array}{ccc} 0 & 0 & 0\\ 0 & 0 & 0\\ 2.95 & 1.82 & -5.25 \end{array}\right]$

$\dot{z}=8\;\mu m/s$	$L=\left[\begin{array}{ccc} 0 & 1 & 0\\ -1 & 0 & 0\\ 0 & 0 & 0 \end{array}\right]$	$A=\left[\begin{array}{ccc} 64 & 2.08 & -70.47\\ -2.07 & 2.61 & -5.73\\ 61.93 & 4.68 & -76.2 \end{array}\right]$

	$M=\left[\begin{array}{ccc} 1 & 1 & -1\\ 1 & 1 & -1\\ -1 & -1 & 1 \end{array}\right]$	$B=\left[\begin{array}{ccc} 0 & 0 & 0\\ 0 & 0 & 0\\ -1.78 & 1.61 & -6.03 \end{array}\right]$

$\dot{z}=40\;\mu m/s$	$L=\left[\begin{array}{ccc} 0 & 1 & 0\\ -1 & 0 & 0\\ 0 & 0 & 0 \end{array}\right]$	$A=\left[\begin{array}{ccc} -6222.65 & -14.78 & 5300.72\\ -61.79 & 1.94 & 41.82\\ -6284.44 & -12.84 & 5342.54 \end{array}\right]$

	$M=\left[\begin{array}{ccc} 1 & 1 & -1\\ 1 & 1 & -1\\ -1 & -1 & 1 \end{array}\right]$	$B=\left[\begin{array}{ccc} 0 & 0 & 0\\ 0 & 0 & 0\\ -64.83 & 0.93 & 44.38 \end{array}\right]$

$\dot{z}=80\;\mu m/s$	$L=\left[\begin{array}{ccc} 0 & 1 & 0\\ -1 & 0 & 0\\ 0 & 0 & 0 \end{array}\right]$	$A=\left[\begin{array}{ccc} -1786.84 & 26.39 & -806.93\\ 301.32 & 3.02 & -314.1\\ -1485.52 & 29.41 & -1121.03 \end{array}\right]$

	$M=\left[\begin{array}{ccc} 1 & 1 & -1\\ 1 & 1 & -1\\ -1 & -1 & 1 \end{array}\right]$	$B=\left[\begin{array}{ccc} 0 & 0 & 0\\ 0 & 0 & 0\\ 12.17 & 1.2 & -38.69 \end{array}\right]$
